# Characterization of Gels and Films Produced from Pinhão Seed Coat Nanocellulose as a Potential Use for Wound Healing Dressings and Screening of Its Compounds towards Antitumour Effects

**DOI:** 10.3390/polym14142776

**Published:** 2022-07-07

**Authors:** Tielidy A. de M. de Lima, Gabriel Goetten de Lima, Bor Shin Chee, Jeferson G. Henn, Yvonne J. Cortese, Mailson Matos, Cristiane V. Helm, Washington L. E. Magalhães, Michael J. D. Nugent

**Affiliations:** 1 Materials Research Institute, Technological University of the Shannon: Midlands Midwest, N37HD68 Athlone, Ireland; a00278595@student.ait.ie (T.A.d.M.d.L.); b.schee@research.ait.ie (B.S.C.); j.henn@research.ait.ie (J.G.H.); y.cortese@research.ait.ie (Y.J.C.); 2 Programa de Pós-Graduação em Engenharia e Ciência dos Materiais—PIPE, Universidade Federal do Paraná, Curitiba 81531-980, Brazil; 3 Laboratory of Genetic Toxicology, Universidade Federal de Ciências da Saúde de Porto Alegre, Porto Alegre 90050-170, Brazil; 4 Embrapa Florestas, Colombo 83410-000, Brazil; mailsondematos@gmail.com (M.M.); cristiane.helm@embrapa.br (C.V.H.); washington.magalhaes@embrapa.br (W.L.E.M.)

**Keywords:** antioxidants, pinhão seed coat, nanocellulose, wound healing

## Abstract

The reuse of agro-industrial waste assumes great importance today. Pinhão is the seed of *Araucaria angustifolia*, which is native to the mountains of southern Brazil, Paraguay, and Argentina. The coat is a by-product of this seed and is rich in phenolic compounds. The present study aimed to use the residue as a precursor material for the production of nanocellulose through the mechanical defibrillation process and perform the characterization of the films and the gel to investigate the effect on the physical and regenerative properties when incorporated with polyvinyl alcohol (PVA). The modulus of elasticity was higher when the MFC of pinhão was added to the PVA. Film and gel had their cytotoxicity tested by MTT assay using 3T3 fibroblast and Schwann cancer cells, and a migration assay was also performed using the scratch test on HaCat keratinocyte cells. For the scratch test, film and gel samples with low concentration presented a complete scratch closure in 72 h. Molecular docking was performed and quercetin had the ideal interaction score values, so it was used with the PACAP protein which presented a slightly moderate interaction with the protein synthesis of Schwann cells, presenting compactness of the compound after 14 ns.

## 1. Introduction

The reutilization of agro-industrial waste assumes great importance today, mainly because of its considerable volume produced in the agri-food industry and its environmental impact. Pinhão is the seed of a native tree commonly known as pinheiro do Paraná (*Araucaria angustifolia*), occurring in the areas of southern Brazil, also on adjacent areas of Paraguay and Argentina. These seeds are highly economic, have a deep cultural aspect, andares nutritionally important, being highly mentioned in current literature due to their importance in food diets [1,2].

**Figure 1:**
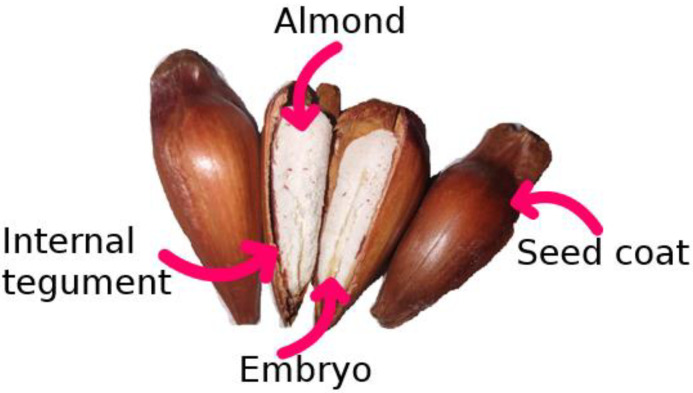
Araucaria seed and its constituents.

The seed coat of pinhão (Figure 1) is a residue of the seed manufacturing process, aimed at food products, and represents approximately 20% of its weight [3]. Pinhão seed coat has low cost and high availability, and is rich in phenolic compounds [4,5], which are acknowledged by industry segments as a driver for potential use due to its high antioxidant activity [6]. Its extract, for example, is a widely studied subproduct and has been used in food packaging, in controlled release formulations containing antioxidant compounds, and protection [7]. In addition, the extract has been reported to be able to inhibit pancreatic lipase, due to the large amount of tannins found within the seed coat [8].

Currently, there are only a few studies in the literature using pinhão seed coat aiming at its pharmacological potential. In this line, Fonseca et al. (2020) evaluated the in vitro cytotoxicity, and in vitro digestibility of aerogels containing pinhão seed coat extract using a C6 rat glioma cells model, in which a reduction of 84% in cell viability occurred (72 h) under a high concentration of extract (3 × 10^−4^ g mL^−1^) treatment [3]. Branco et al. (2015) reported the antiproliferative activity of an extract obtained from seed coat on human laryngeal carcinoma (HEp-2) and non-tumour (HEK-293) cell. Along with the aforementioned work, the chemical composition of extract demonstrated selective cytotoxicity and pro-apoptotic activity in tumor cells, such as HEp-2 [4] and breast adenocarcinoma (MCF-7), pulmonary carcinoma (NCIH460), cervical (HeLa) and hepatocellular carcinoma (HepG2), presenting different inhibitory activity depending on the extraction approach used for the seed coat [5]. 

The development of films with high phenolic compounds, so as to obtain antioxidant properties or active packaging materials, has been commonly investigated as well for pinhão seed coat [6]. Cruz et al. (2021) suggested the use of these extracts as active packaging containers in which the compounds migrate to the food, improving sensory characteristics, increasing the shelf life, prevent chemical and microbiological degradation, and ensure food safety [7]. Additionally, Spada et al. (2018) investigated the effect of a macro pinhão seed coat powder in starch films and used various chemical and physical treatments in order to better improve these materials’ interaction. These treatments were not successful at presenting an homogeneous film, with visible powder aggregation and film non-uniformity while also presenting low mechanical properties required for food packaging, but with an increase in water vapour permeability [8].

In addition, besides the aforementioned works that used the extract of the seed coat, and not the pure seed coat, the extraction limits the components obtained in the seed coat and its availability. Therefore, usage of the pure seed coat without limiting the availability of the nutrients may be achieved by defibrillating the cellulose contained within the coat, obtaining a nanocellulose or a nanosuspension.

Nanocellulose is formed by mechanical defibrillation that grinds cellulose bundles into a reduction of its size in fibrils on the order of nanometres (0.1 and 100 nm), producing a gel-like characteristic [9]. The usage of nanocellulose obtained from the seed coat of pinhão has been previously reported by our group, in which a novel high-functional cereal bar was developed, which presented twice the amount of fibers than commercial cereal ones, with high antioxidant activities [10].

The usage of films for nanocellulose using by-products has been demonstrated previously using bleached kraft pulp with tannins and yerba mate [11,12], which reports that these materials present a good electrostatic interaction with cellulose, maintaining its antioxidant activities with a wide range of applications [13]. Another option suggested in this work was the use of polyvinyl alcohol to form the film, such as cellulose/PVA hydrogels that exhibited homogeneous porous structures with good miscibility [14]. The study of active pharmaceutic ingredients within a polymeric system presents important advances for nanostructured devices [15,16]. 

This study investigated the usage of pinhão seed coat via mechanical defibrillation in order to form nanocellulose, and the derived nanocellulose suspension was used to produce a more homogeneous film with high mechanical properties. In addition, pinhão seed coat gel suspension and films were tested for in vivo applications using cell tests as its viability and scratch test. These cell viability and scratch tests have not yet been reported in the literature for pinhão.

## 2. Materials and Methods

### 2.1. Materials

Rat neuronal Schwann RT4-D6P2T, mouse fibroblast NIH 3T3, human HaCat embryo cells, and bacterial *Escherichia coli* 25922 strain were purchased from American Type Culture Collection (Manassas, VA, USA) and *Staphylococcus aureus* 12981 strain was purchased from National Collection of Type Cultures (Colindale, London, UK). Pinhão seed coat from Araucaria tree for microfibrillated cellulose (MFC) preparation, and polyvinyl alcohol (PVA, average Mw 146,000–186,000, 87–89% hydrolysed) was purchased from Sigma-Aldrich (St. Louis, MO, USA). Cell culture media and supplements were purchased from GIBCO™sourced from Thermo Fisher Scientific (Waltham, MA, USA). 

### 2.2. Micro-Fibrillated Cellulose (MFC) Preparation

**Figure 2:**
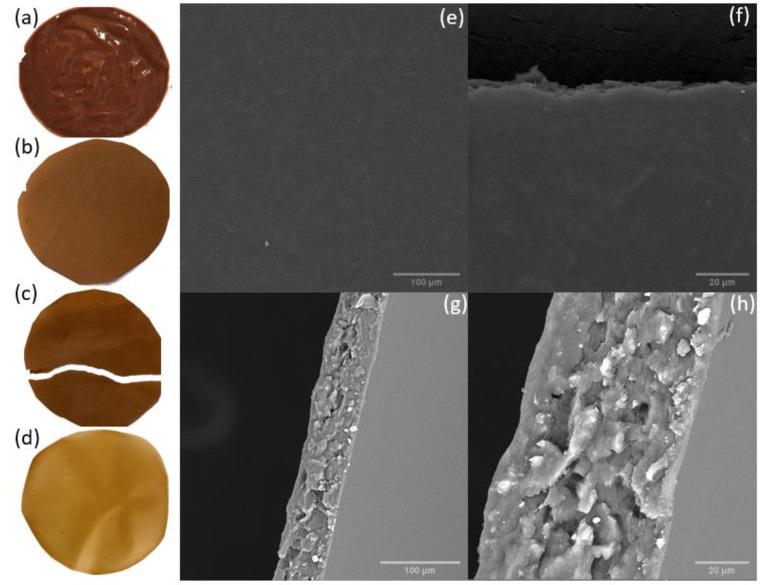
(**a**) MFC gel suspension of pinhão seed coat 6% wt%/v with gel-like characteristics; (**b**) a film produced using the gel suspension and water using the sieve method, dried overnight at 60 °C; (**c**) demonstration of how the film is brittle and (**d**) film produced using MFC gel suspension of pinhão seed coat 1% wt%/v with 5% of PVA by casting method, dried overnight 60 °C. Scanning electron microscope images for surface (**e**) 500×, (**f**) 2000× and cross-section (**g**) 600×, (**h**) 2000× for films produced using MFC gel suspension of Pinhão seed coat 1% wt%/v with 5% of PVA by casting method.

Initially, a suspension was prepared of pinhão (6%, *w*/*v*) using distillate water, then was fragmented using a 450 W blender for 10 min. Subsequently, it was subjected to further grinding using a supermasscolloider, Masuko Sangyo Microfluidizer (Masuko Sangyo Ltd., Kawaguchi, Japan), using a rotation of 1500 rpm, 30 steps and distance between discs of 0.1 mm. In this process, the fibers are reduced to the nanometric size (0.1 and 100 nm) and through the friction between the stones, the abrasive forces are induced on the cellulose. With this procedure, it is possible to obtain a nanosuspension formulation that exhibits a gel-like characteristics. With this gel of pinhão seed coat, a film was synthesized using the sieve method from the gel suspension (Figure 2a) described by [17], and using this method is possible to make a film through the entanglement of the fibers through the filtration (Figure 2b).

### 2.3. PVA Solution and Film Formation

PVA films with pinhão gels were prepared using solution of PVA + gels suspension + water resulting in films with 5% (*w*/*v*) of PVA and 1% (*w*/*v*, 0.01 g mL^−1^) of gels of pinhão. Afterwards, 20 mL of this solution was poured on petri-dish and dried at 60 °C overnight using an oven (Gallenkamp hot box oven with fan, size 1, A. Gallenkamp & Co., Ltd., London, UK) as a solvent-cast methodology. The samples used in this work follows the denomination pure pinhão seed coat, film PVA and film PVA/pinhão.

### 2.4. Scanning Electron Microscopy (SEM)

The structures of the PVA pinhão film surface and cross-section were observed using a Mira scanning electron microscope (TESCAN Performance in Nanospace, Tescan Orsay Holding, Brno, Czech Republic) with the back-scattered electron (BSE) mode. Prior to imaging, specimens were sputtered with gold in Baltec SCD 005 for 110 s at 0.1 mbar vacuum yielding a coating of ca. 110 nm. The images were recorded at an acceleration voltage of 20 kV and a magnification range of 600–2000. The film produced was cryofractured for the cross-section so this internal structure conforms to the guided orientation within the film.

### 2.5. Fourier Transform Infrared Spectroscopy (FTIR)

PerkinElmer Spectrum One Fourier transform infrared spectroscopy (FTIR) (PerkinElmer, Waltham, MA, USA) was used to investigate the existence of chemical interactions between the films of PVA and pinhão. The IR spectra were recorded in the spectral range of 4000–500 cm^−1^.

### 2.6. Thermo and Mechanical Analysis

For differential scanning calorimetry (DSC), film of PVA, pinhão and PVA/pinhão were weighted around 4–5 mg and encapsulated in alumina sample pans. A temperature ramp from 20 °C to 600 °C at a rate of 10 °C/min was used with an empty closed alumina pan as a reference. The experiments were carried out under nitrogen flow of 50 mL min^−1^, in a Q600 SDT—TA Instruments (TA Instruments, New Castle, PA, USA).

Dynamic mechanical analysis (DMA) analyses were performed on DMA Q800 TA Instruments (TA Instruments, New Castle, PA, USA) equipment using the film tension clamp. Stress-strain tests with ramp force of 1 N/min up to 18 N. All tests from DMA were performed using three scans per sample. The mean stress−strain for each film condition was used for the graph.

### 2.7. Swelling Test

For swelling measurement, pure PVA films and PVA/pinhão films were measured gravimetrically. After that, the samples were immersed in distilled water to measure the swelling kinetics. The samples were retained from water for 1, 2, 4, 6, 8, 24, and 48 h. The excess surface solution was gently removed with a paper towel and the swollen samples were weighed. The percentage of the dilation ratio of a hydrogel was calculated and plotted against time.

Besides, the second-order equation proposed by Schott for swelling data was carried out to do the kinetic model [18]:

(1) $$\frac{t}{W}=A+Bt$$

In this equation, *B* = *W*_∞_^−1^ and *A* = (d*W*/d*t*)^−1^, the water absorption capacity at time t and at equilibrium are represented by *W* and *W*_∞_ respectively. The opposite of the theoretical equilibrium swelling is *B* and the opposite of the initial swelling rate is *A*. The angular and linear coefficients, (*W*_∞_ and *Ks*) are possible to obtain plotting *t*/*W*; the swelling rate constant is *Ks* (g/g min) and is associated by *Ks* = (A*W_∞_^2^)^−1^.

### 2.8. Pinhão Bioactive Compounds Release

Release was performed in DI water at 37 °C. For this, 0.100 g of film was added to 4 mL of DI water. The released concentration was determined by UV-Vis spectroscopy (UV-VIS Spectrophotometer 1280, Shimadzu Corporation, Kyoto, Japan). This was performed with the help of a multivariate calibration curve, built based on absorbance, in the range 900 to 330 nm, from pinhão extract solutions with concentration in water ranging from 0.037 to 0.37 mg mL^−1^ achieving a calibration curve with internal validation data of R² = 0.998 and standard error = 7.2 × 10^−3^, for one factor.

### 2.9. Antibacterial Evaluation

The antibacterial activity of the samples was investigated by Kirby Bauer disk diffusion assay (Hardy Diagnostics, Santa Maria, CA, USA) and a microdilution minimum inhibitory concentration (MIC) assay. For both tests, a range of two-fold dilutions was used from the neat solutions down to 1:128. MIC test was performed using the microdilution MIC test and used resazurin as a bacterial viability indicator. For the disk diffusion, samples were cut into 6 mm discs which were then sterilized by UV light. Mueller−Hinton agar plates were inoculated with 100 µL of either *E. coli* or *S. aureus* at a density of 1 × 10^8^ CFU mL^−1^ which was dispersed over the entire agar surface with a sterile glass L-shaped spreader. Triplicate discs were aseptically placed on each plate before incubation at 37 °C overnight. After incubation, the diameter of each zone of inhibition observed was measured and recorded.

### 2.10. In Vitro Cytotoxicity Assay

For the cytotoxic evaluation, both gel and film were tested. Films were sterilized using ethanol 70% for 30 s, phosphate buffered saline (PBS) for 30 s, followed by DMEM media for 30 s prior to cytotoxic evaluation following the methodology of a previous report [12,19]. MTT colorimetric assay was used to evaluate the cytotoxicity potential of the gels and films. Both Schwann cells and 3T3 cells were incubated at 37 °C and in a 5% CO_2_ incubator. Cells were then exposed to different concentrations of gels and films for 24 h.

For the gels, the concentration tested were 0.005%, 0.01%, 0.05%, 0.075%, 0.1% and 0.2%; and for the films, these were 0.057%, 0.12%, 0.14%, 0.17%, 0.23% and 0.26%. Concentration values for the film were obtained from the calculated release test. Briefly, after treatments, cells were washed with PBS before the addition of 100 μL serum-free media containing yellow tetrazolium salt dye (1 mg mL^−1^) and incubated for 3 h at 37 °C. After incubation, the supernatant was removed, the residual purple formazan product solubilized in 200 μL dimethyl sulfoxide (DMSO), and its absorbance measured at 570 nm (BioTek Synergy HT, Swindon, UK). The absorbance of the negative control was set as 100% viability and the values of treated cells were calculated as percentage of control.

### 2.11. In Vitro Cell Migration Test

Cell-based scratch assay was performed according to Ko (2011) and Sperotto et al. (2018). HaCaT cells had up to 90–100% confluence of the filled base in a 6-well culture plate for 24 h. Scratching was performed using a 200 µL micropipette tip [20,21]. After the scratch, the cells were washed with PBS and then the gel and film were added. The gel was tested at concentrations of 0.2% and 0.005% and the film at concentrations of 0.25% and 0.029%. The compound used as a negative control was the fresh DMEM serum-free medium, and, as the positive control, fresh medium with 10% fetal bovine serum (FBS). Images were obtained immediately (0 h) and after 2, 4, 8, 12, 24, 48, and 72 h of the test. The scratched areas were measured using the ImageJ^®^ software (National Institutes of Health, Bethesda, MD, USA). The healing was plotted versus time after scratch.

### 2.12. Pre-Processing of Molecules Prior to Simulation

The 3D structure of the PAC1 human receptor was obtained from the Protein Data Bank (http://rcsb.org, accessed on 15 June 2022), under the code 6LPB. This protein was then prepared using the CHARMM-GUI application, the first part was to consider only the PACAP and PAC1 segments of this protein. The protein then is manipulated to include missing residues and disulphide bonds [22].

The compounds used in this work are derivatives found in pinhão seed coat. These compounds were obtained from zinc15 database [23] namely beta-sistosterol (ZINC568758129), campesterol (ZINC568877419), catechin (ZINC169542954), guaiacylglycerol (ZINC175016828), oleic acid (ZINC170248642) and quercetin (ZINC181051096).

These compounds were first pre-processed using Avogadro software to minimize energy using MMFF94s force field followed by optimization using a semiempirical PM7 method within MOPAC2016 software [20].

### 2.13. Docking Simulation

The docking methodology was performed using AMDock software [21], in order to start the docking the input files were prepared. Protein was transformed from *.pdb files to *.pdbqt where the heteroatoms and alternates were removed, and protonation states were added while also merging charges and removing non-polar hydrogens. For ligand, protonation states, merge charges and remove non-polar hydrogens using a pH 7.4 [24] with AMBER Force Field. A search space was defined, blind docking, using the AutoLigand tool [25].

Docking was then performed using AutoDock Vina tool [26] with 4 CPU usages, and exhaustiveness of 8 and 10 number of poses.

After docking, the compounds with the lowest values of affinity and efficiency were selected in order to present the results. To illustrate the binding affinity with each compound, ChimeraX software (Resource for Biocomputing, Visualization, and Informatics, San Francisco, CA, USA) was used [27].

### 2.14. Molecular Dynamics Simulation

For molecular dynamics, the quercetin compound was used in order to understand the interaction with the protein. First, the protein and compound were combined within a single file, thereafter the topology files were produced using the solution builder from CHARMM-GUI with CHARMM36m force field. Solvation of the protein+compound was performed using a rectangular shape with CHARMM specifying the size of the box based on molecule size, ions were added with KCl basic ions with a concentration of 0.15. Afterwards, a grid for PME FFT was generated automatically and produced files for OapenMM that were used for the simulation with an NPT ensemble at 303.15 K for dynamics input.

Molecular dynamics was performed using a python script developed by Arantes et al. (2021) [28] which utilizes the google collab feature to run the google compute engine backend for this simulation using the OpenMM molecular dynamics code [29]. Briefly, the inputs from the CHARMM-GUI solution builder were used and the system was equilibrated with 100 ps with an integration timestep of 2 fs at 303.15 K with 1 bar of pressure with a position restraint containing a constant force of 500 KJ/mol. Lastly, the MD simulation containing NPT ensemble was performed, using a time of 15,000 ps, with integration timestep of 3 fs using at 303.15 K with 1 bar of pressure. The trajectory was aligned and the RMSD of the system along with its 2D RMSD, radius of gyration, and PCA as distribution were plotted.

## 3. Results and Discussions

General characterization of pinhão seed coat was performed by the group previously, using the same concentration [30]. The film produced without PVA became brittle and the shape of the film lacked resistance, this is seen by the macroscopic photographic images of the samples (Figure 2c) to exemplify the brittleness of the film after handling it gently. When bleaching is previously performed on the husk, one obtains a bleached pulp of cellulose [30]. With such material, it is possible to make films with up to 5 GPa of elastic modulus, but as the idea of this work is to use the compounds found in the husk, therefore, no pre-treatment was carried out, so the film obtained from MFC’s unbleached husk was brittle. However, the film made with PVA after solvent-casting presented a homogeneous visual morphology without aggregation of particles and it is malleable (Figure 2d). Therefore, it was the methodology used for the following tests.

### 3.1. Scanning Electron Microscopy (SEM)

The film with pinhão seed coat and PVA surface and cross-section was analyzed using SEM to observe the morphology structure of these materials (Figure 2e–h). The surface of the film was smooth, this side was the one in contact with the petri dish when drying. Nonetheless, bundles of cellulose can be seen in high resolution which may be due to the phenolic interaction of the seed coat with the MFC and PVA, as it has been reported that both materials can bind quite well with phenolic compounds [11]. In the cross-section (Figure 2g,h), it is possible to observe the lamella structure was similar to a previous work of our group which uses bleached eucalyptus nanocellulose with polyvinyl alcohol hydrogels [14].

### 3.2. Fourier Transform Infrared Spectroscopy (FTIR)

**Figure 3:**
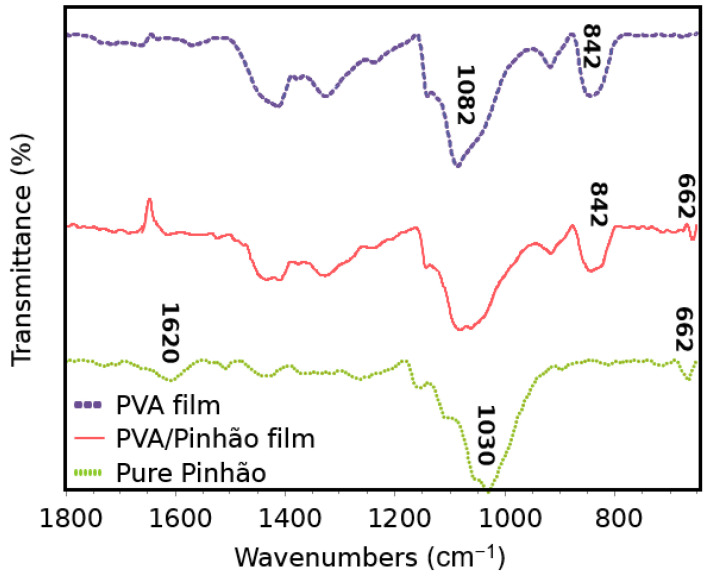
FTIR for PVA film, PVA/Pinhão film and Pinhão pure film.

The infrared spectra analysis was performed on pure PVA, pure pinhão seed coat nanocellulose gel suspension, and in the film to see the interaction between these components (Figure 3). The majority of the bands from cellulose and PVA were already previously described and are widely known [14].

The peak observed at 1620 cm^−1^ was assigned to the C=C ring stretch of aromatic rings, this was reported previously and assigned due to the rich tannins found in the seed coats [31]. Several bands between 1319 and 1030 cm^−1^ in pure pinhão can be attributed to C-O bonds of phenols. This shape of several bands is characteristic of different types of C-O bonds in different phenolic groups, indicating the presence of tannins in the pinhão coat [10]. A result of 1158 cm^−1^ is attributed to the asymmetric stretching of cellulose and hemicellulose C-O-C and in 995 cm^−1^ were characteristic of alkene groups for C-H bonding [8,32]. The small peaks at 662 cm^−1^ in the PVA/pinhão film and in the pure pinhão samples were attributed to C-OH out-of-plane bending [33].

### 3.3. Thermal and Mechanical Analysis

**Figure 4:**
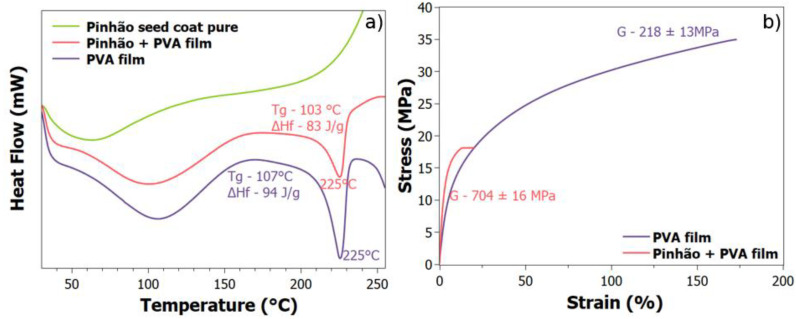
(**a**) Differential scanning calorimetry for pinhão seed coat pure, PVA film and Pinhão/PVA film; (**b**) Stress-strain curves for the PVA film and Pinhão/PVA film.

Figure 4a shows DSC curves of Pinhão seed coat pure, PVA film and PVA/Pinhão films. The PVA exhibits an accentuated endothermic curve corresponding to the melting of the crystalline phase of the PVA associated with the pre-thermal events of this material. The Tm values remain approximately constant for the PVA film and the composite. The heat of fusion varied when nanocellulose was incorporated into the PVA, this decrease of ΔH*_f_* and T*_g_* for films with PVA/Pinhão were also found by Mihaela et al. (2011) who studied the properties of polymer composites with MFC [34]. Therefore, it is possible to deduce that the blend of PVA and Pinhão seed coat presented a lower crystallinity degree. It is possible that PVA chains may blend it better with MFC groups rather than on its own, which depends on the percentage of cellulose microfibrils presented in the gel [14].

Examining the DSC of PVA film and the composite, it is possible to identify a relaxation in the region between 100–150 °C. This relaxation is known as β*_c_* because of the relaxation of the domains in the crystalline regions of the PVA and was found in the literature [35] not seen in the pure pinhão seed coat. This T*_g_* value is also decreased, another confirmation of the polymeric chains now arranged within the cellulose fibrils.

The mechanical properties of the PVA film and PVA/pinhão films are summarized in Figure 4b. In the curve of the PVA film, the elasticity of this film can be observed. Adding nanocellulose to PVA films increases the strength of the film; however, it presents a loss in elasticity. Films produced with microfibrillated cellulose are very resistant in tensile tests [14,17]. The values of the modulus of elasticity of MFC films with bleached kraft pulp from *Eucalyptus* sp. can be found in the literature with a value of up to 5 GPa [36]. In this work, the modulus of elasticity was 218 ± 13 MPa for PVA film and 704 ± 16 MPa when MFC of Pinhão was added in the film. The value found in this work is lower because the PVA may alter the structure of the nanocellulose and these chains are not as well distributed as a pure nanocellulose film, but at the same time, it gains elasticity and transparency [37].

### 3.4. Swelling Test

**Figure 5:**
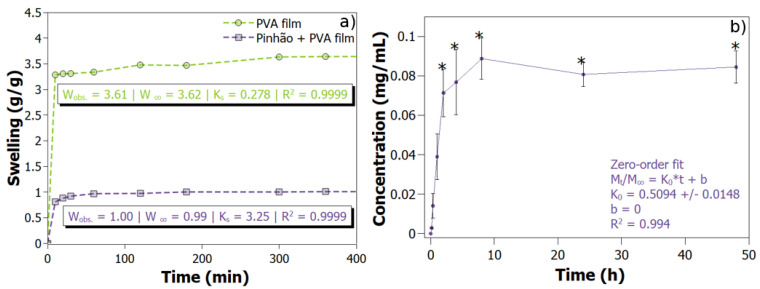
(**a**) swelling ratio of the studied for PVA films and for PVA/pinhão films; (**b**) Drug Release PVA/pinhão film, concentration (mg extract mL^−1^), zero-order model with R^2^ prediction: 0.994, with a prediction error of 7.2 × 10^−3^ at water, 37 °C. All data points are plotted as the mean ± SD (n = 3). Treatments followed by * in each concentration do not differ by Tukey’s test (*p* < 0.01).

Swelling tests were carried out for the pure PVA film and PVA/pinhão film. Values for the swelling model were obtained for K (swelling rate) and *W*_∞_ (maximum theoretical absorption value), which together with the Wobs value (maximum experimental absorption value), are shown in the graph (Figure 5a). There is a decrease in swelling, when adding the MFC of pinhão, and such behaviour is expected since there are more interactions between PVA and MFC, making it difficult for water to penetrate the polymer (de Lima et al., 2020). The pure PVA film has a higher swelling value than the film with pinhão because this polymer is a linear chain that, in the presence of water, allows the formation of intermolecular and intramolecular hydrogen bonds. Previous works evidenced that the addition of nanocellulose to PVA alters the binding mechanism, whereas the self-association within the crosslink in a pure PVA decreases to bind within the cellulose backbone. In addition, PVA synthesis with other known compounds that alters the crosslinking behaviour also causes a decrease in the hydroxyl groups available [38]. Results found in the literature confirm that the addition of MFC reduces the affinity of PVA with water, leading to a strong reduction in the swelling. Consequently, increased swelling rate, and the addition of 5% and 40% of MFC exhibited, in a previous work, a 7% and 50% reduction in swelling, respectively compared to neat PVA [39].

### 3.5. Drug Release

The drug release (Figure 5b) was the best-fitted model by the zero-order model. Different kinetic models of drug release were applied and interpreted in the form of graphical presentation and evaluated by the correlation coefficient (R^2^). The highest degree of correlation coefficient determines the most probable model outcome for the mathematical model that determines the pinhão release kinetics in the film with the PVA in water. The sample showed a good fit to the zero-order release model (R^2^ prediction: 0.994, with a prediction error of 7.2 × 10^−3^), which is based on the slow release of the active substance from dosage forms that do not disaggregate. This zero-order model is ideal for biomedical and pharmaceutical applications as it is known to present a concentration of active ingredients within non-toxic plasma concentrations. However, the release was very fast—within 2 h a profile typical of fast drug release. The bioavailability of a bioactive compound is improved with a timely and targeted release [40]. In the literature, there are several works that use the same model proposed herein for the release of phenolic compounds. A previous work used the zero-order model for the release of tyrosol—a phenolic antioxidant [41].

### 3.6. Antibacterial Evaluation

For antimicrobial activity, only the suspension of nanocellulose from the pinhão seed coat was evaluated. The gel did not inhibit the growth of Gram-positive bacteria *S. aureus*. In addition, it showed no activity against Gram-negative *E. coli* bacteria. For the MIC test, no reduction in bacterial viability at any concentration was found with either *E. coli* or *S. aureus*. Finally, the disk diffusion test also presented no zone of inhibition with either sample at any concentration with both tested bacteria. Trojaike et al. (2019) found that the pinhão seed coat extract inhibited the growth of *S. aureus*, *Bacillus cereus*, *Listeria monocytogenes*, *Listeria innocua*, and *Aeromonas hydrophila* after thermal treatment [42]. Additionally, it has been reported that pinhão seed husk extract is not able to inhibit Gram-negative bacteria [43].

It is possible that the gel at this concentration was not able to provide enough phenolic compounds that are known to inhibit these bacteria. Nonetheless, it is important to remind that phenolic within the seed coat may have deeply interacted with PVA so their active sites of ability to inhibit the bacteria may have been hidden and could explain the rise in the mechanical property and in the tests found herein. However, techniques for improved synthesis of nanoscale materials for compound delivery do exist albeit are time-consuming and very specific to each polymeric carrier [15].

### 3.7. In Vitro Cytotoxicity Assay

**Figure 6:**
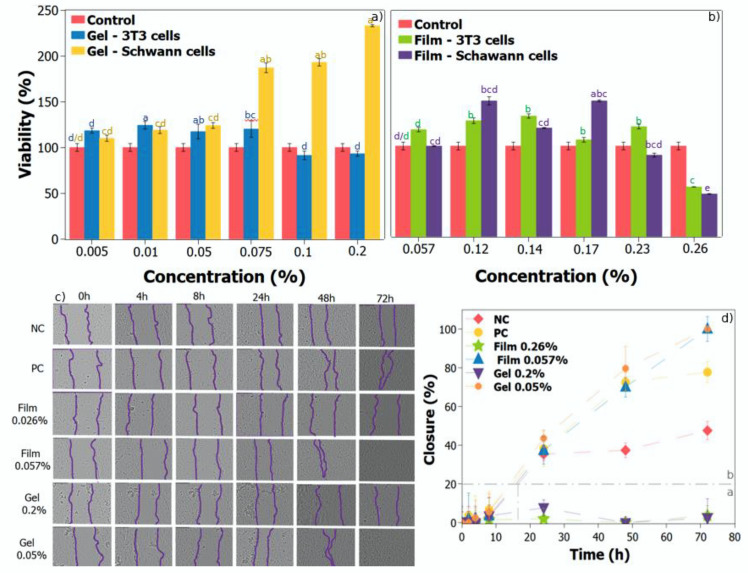
In vitro cytotoxicity assay (**a**) samples tested with 3T3cells and Schwann cells to the gel of Pinhão; (**b**) samples tested with 3T3cells and Schwann cells to film of Pinhão/PVA.; (**c**) Scratch assay in HaCaT cells over 72 h (**d**) results are expressed as mean ± standard error median of three independent experiments performed in triplicate. Medium with 10% FBS was used as positive control (PC) and medium without serum was used as negative control (NC); the measurements were performed considering the area of the scratch considering time 0 of each treatment with 0%. In (**a**,**b**) letters within the same color represent the Tukey post-hoc treatment for each specific test (**a**–**d**), samples that have the same letter are statistically equal, (blue—3t3 cells in gel, yellow—Schwann cells in gel) (*p* < 0.05). Control represented by another color is also compared statistically. Samples with the same letters within each color do not differ statistically. For (**d**) samples that were in region, b were statistically significantly different compared to samples in region (**a**).

Cell viability was performed in 24 h treatments for both pinhão gel and PVA/pinhão film using 3T3 fibroblast and Schwann tumor cells (Figure 6a,b). Regarding the gel, 3T3 cells viability was significantly higher than the negative control in the lowest concentrations (0.005%, 0.01%, 0.05%, and 0.075%), whilst concentrations of 0.1% and 0.2% decreased cell viability when compared to the negative control (approximately 90% of viability for both concentrations). In Schwann cells, the highest the concentration of the gel, the highest the viability, with a maximum of 233% at the concentration of 0.2%.

For films, 3T3 cell treatments showed the highest viability at 0.14% concentration, with a value of 133%, and the highest concentration (0.26%) significantly decreased cell viability to 55% when compared to the negative control. In Schwann cells, the highest viability (150%) was observed at the concentration of 0.17%. On the other hand, the highest concentration (0.26%) had a significant cytotoxicity of 47%. In general, and according to previous MTT assays, viability values below 70% can be considered toxic for both cell lines [12,44,45].

There is a lack of scientific literature on the usage of pinhão seed coat in cellular activity. Mostly, the literature cites the usage of the extract, for example, Oliveira et al. (2021) evaluated the cytotoxicity of extracts for breast adenocarcinoma (MCF-7), pulmonary carcinoma (NCIH460), cervical (HeLa), and hepatocellular carcinoma (HepG2) for pinhão seed coat and extract (cooking water) whereas catechin was the principal component and the seed coat extract presented higher antioxidant capacity and increased inhibition of human and porcine α-amylases while being non-toxic to normal cells. However, the pinhão seed coat extract presented cytotoxicity to non-small cell lung, cervical, hepatocellular, and breast carcinoma cell lines [5]. A previous work from Fonseca et al. (2020) produced aerogels with pinhão coat extract using native and anionic starches as carrier matrix in order to evaluate its effect on cell viability, in vitro cytotoxicity, and in vitro digestibility with C6 rat glioma cells, presenting cell viability reductions up to 50% for the free extraction from pinhão within concentrations of 100, 200, and 300 µg mL^−1^ [3]. In our work, the concentration tested was much lower than those used in the literature, and even so, it showed high cell viability compared to the control.

### 3.8. In Vitro Cell Migration Test

Cell migration assay was performed by using the scratch test method in HaCat keratinocyte cells, and the maximum and minimum concentrations of the MTT assay were used (Figure 6c). After 72 h of treatment, the negative control showed 45.5% of closure, while the positive control closed 77.3%. After 24 h of treatment, film and the gel lower concentration samples presented closure values of 37.36% and 43.57%, respectively. These values increased to 70.4% and 79.67% after 48 h, reaching complete closure in 72 h (Figure 6d). Contrarily, both gel and film samples did not show a significant difference in closure after 72 h of treatment with their highest concentrations, corroborating MTT film results, which showed a decrease in cell viability for both 3T3 and Schwann cells, not allowing the cells to properly migrate. Regarding the highest concentration for the gel sample, even though the MTT assay has not shown a decrease in cell viability, there was no scratch closure for this group. The same behavior in the in vitro cell migration test was observed by Csepregi et al. (2020) for plants containing phenolic groups. The less concentrated leaf extracts produced the most efficient stimulation of cell migration, when compared to the extracts with higher concentrations [46]. Prado et al. (2020) tested seed and peel extract of araticum which contained larger quantities of phenolics, for the seed extract with the lowest concentration the closure was 73% while for the highest concentration the closure was 3.8%. The ability of peel extract components to prevent cell migration may explain the inhibitory action seen in a scratch assay. The low concentration of araticum extract (3.6 µg mL^−1^) could promote wound healing, while a high concentration (36 µg mL^−1^) would be useful in the control of hyperproliferative epidermal diseases, as it had a cytostatic effect on HaCaT cells [47].

### 3.9. Pinhão Seed Coat Component

The pinhão seed coat is composed of many components such as organic acids, furans, phenols, sterol, organic acids like malic acid, azelaic acid, succinic acid, catechol, and sugar acids like glyceric acid, arabinonic acid, arabitol, xylitol among others [30]. Many of these compounds have positive results in the treatment of tumors, such as catechin, campesterol, quercetin, oleic acid, and beta-sistosterol (Table 1). It is important to describe each of them in order to understand the effect of each compound, while they may also have synergism within each other, not yet thoroughly studied. A discussion of the values found within the literature and the compound effect, as well as the EC_50_, are described in Supplementary Material S1.

### 3.10. Molecular Docking

In order to deepen the understanding of the pinhão films behavior towards Schwann cells, the compounds found within nanosuspension of pinhão seed coat, which presents a potential screening for antitumor effect, were used to evaluate their interaction between PACAP protein. This protein is expressed in peripheral nerves and their levels are increased following axotomy; also, when PACAP interacts with the PCA1 receptor, it stimulates tPA expression and proteolytic activity in Schwann-cell-like cultures [62]. In addition, PACAP is related to cellular survivability, which prevents Schwannoma into enter apoptosis [63]. For tumor cells, apoptosis mechanisms are often inhibited in order to allow the proliferation and growth of the tumor. This same protein is important for cell myelination which in turn, is important for its own protection and PACAP does this via activation of a signaling pathway labelled as PI3k/Akt, which in many cancers is overexpressed and related to tumor resistance [64].

**Figure 7:**
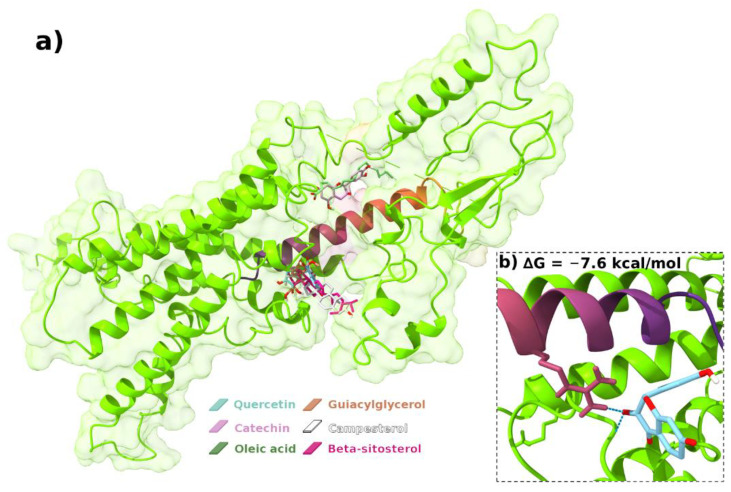
(**a**) Predicted bonded interactions (green dashed lines) between quercetin, catechin, oleic acid, guiacylglycerol, campesterol, and beta-sitosterol interact via H-bonding with PACAP and PAC1; (**b**) binding mode for the catechin and quercetin with the highest interaction for molecular dynamics with the protein.

Therefore, PACAP with PAC1 receptor was used to investigate if any interaction between this protein and Pinhão compounds occurs, the ones that are known to be antitumor. Molecular docking (Table 2 and Figure 7a) presents that all investigated compounds have two forms of interaction with the protein, and two binding sites (Table A1 in Appendix A. The first one is where the majority of Pinhão compounds are included and present compounds that interact via H-bonding with PACAP and PAC1, for Quercetin and Guaiacylglycerol, while the other components campesterol and beta-sistosterol have a weaker interaction without any H-bond and only active van der Waals forces (Table A2 in Appendix A). Alternatively, oleic acid and catechin were coupled for the other binding site presenting lower interactions with the protein. From these results, it can be noted that both hydrophilic and lipophilic compounds are present in the studied binding sites and the overall net charge weakly influences the binding order.

The values for the obtained docking sites relate that both catechin and quercetin have the highest interaction with the studied protein, meaning that hydrophilic compounds in this case present a higher interaction with the lowest efficiency values. The inhibition constant (Ki), as well as the docking energy, predicts information about the stability of the formed receptor-ligand complex, the lower Ki, the greater the stability of the complex and, consequently, the greater the effectiveness of the ligand in inhibiting the receptor [65]. The KI units are in µM. Overall, molecules with multiple H-bond pharmacophores, can be promising as an inhibitor against Schwann cells. Considering the values of the molecules towards docking, the compound with the highest interaction was used for molecular dynamics with the protein, related to quercetin that presented the highest negative score values (Figure 7b) and formed H-bonds with significant residues, suggesting that they may form stable interactions with the protein causing the inhibitory effect.

**Figure 8:**
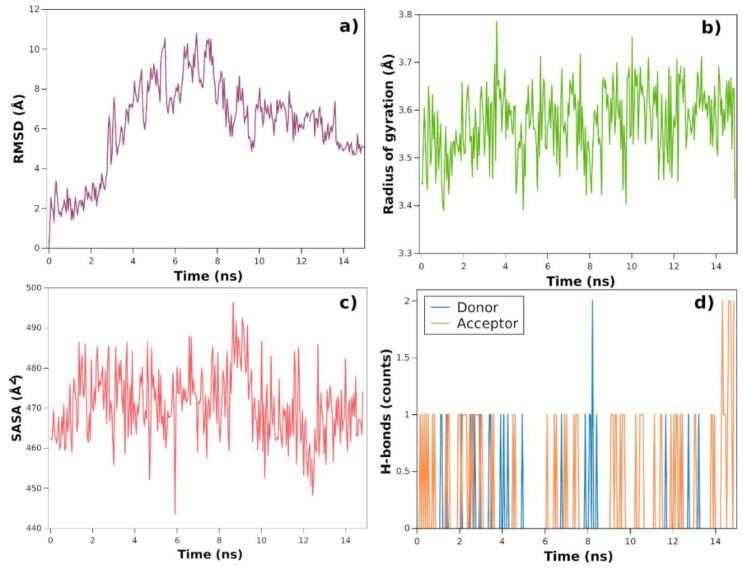
Molecular dynamics analysis using RMSD values over time (**a**), the radius of gyration over time (**b**), solvent-accessible surface area over time (**c**), and number of H-bonds whether protein as donor or acceptor (**d**).

After targeting the best result from the docking simulation, quercetin was submitted to 15 ns of MD simulations to observe their behaviour towards the targeted protein (Figure 8). The system presents that stabilization is reached after 14 ns with RMSD values of ~0.5 nm (Figure 8a), signifying that this compound vibrated with increased frequency over time and had a slightly poor accommodation inside the PACAP+PAC1 complex. The radius of gyration during simulation time presents values of ~3.5 nm (Figure 8b), with a slightly decreased variation on the values after reaching 14 ns; therefore, exhibiting that quercetin is accommodated throughout the whole simulation and presents the protein compactness. A similar profile is seen for the solvent-accessible surface area (SASA) (Figure 8c), related to the water contact surface between the ligand and protein, which also presented low values confirming the binding aspect. Ideally, a simulation between with and without the quercetin should have been made to compare the SASA values and understand the effect of this compound. Nonetheless, the values are stable indicating no significant changes in the protein structure. Finally, the number of H-bonds was computed and presented the same response found prior to the simulation (Figure 8d and Supplementary Material S1) with only one binding mode. However, as the compound adapts to the system, there are two H-bonds found, considering the protein as an acceptor of H-bonds.

Overall, the system relates that there is an interaction between quercetin with PACAP+PAC1 protein, more specifically MD simulation suggests that there is a possibility that quercetin in this case can interact with the PACAP protein and inhibit its activity by blocking the pathways of gene expression for Schwann cells production.

Studies on docking using quercetin have reported applications in many areas. Adeoye et al. (2019) evaluated the inhibitory activity of quercetin and apigenin using in silico docking studies, which presented binding energies ranging between −6.5 kcal/mol and −7.1 kcal/mol by interacting with protein Calcium ATPase SERCA and CAX [66]. Maharani et al. (2020) evaluated by docking analysis the potential of single bulb garlic flavonoids (quercetin, isoquercetin, and kaempferol) in inhibiting lanosterol synthase. Binding affinity values of −9.8kcal/mol from quercetin showed a potential ligand to treat hypercholesterolemia [67]. Gu et al. (2021) evaluated the potential effects and mechanisms of quercetin on COVID-19-induced AKI by network pharmacology. Molecular docking was applied to verify and test how Quercetin interacts with target proteins 3CL (PDBID: 6LU7) and ACE2 (PDBID: 1R42). The binding energy of Quercetin with the target proteins ACE2 and COVID-19 main protease 3CL were −3.78 and −4.53 kcal/mol respectively [68].

## 4. Conclusions

The production of nanocellulose by mechanical process allowed us to obtain more homogeneous films than those found in the literature. Mechanical tests by DMA analysis exhibited that the addition of pinhão nanocellulose increases the film strength. The addition of nanocellulose in PVA significantly increased the value of the elastic modulus, from 218 ± 13 MPa for the PVA film to 704 ± 16 MPa when added MFC is added. Due to stronger intramolecular and intermolecular bonds, equilibrium swelling decreased (72%) leading to a faster swelling rate (~12 times) with the addition of MFC from the pinhão seed coat. Cell viability assay revealed that the best results for 3T3 cells were when the lowest concentration samples for both gel and film were used (≤ 0.075%). On the other hand, gel tested in Schwann cells showed that the highest the concentration, the highest its viability (achieving higher than 200% of viability for 0.2% of concentration). However, the film with a concentration of 0.26% presented significant toxicity to both 3T3 and Schwann cells (<70% of viability), making him a candidate for further studies regarding its cytotoxic mechanisms, possibly using a different approach for targeted release. The scratch test revealed that the most concentrated samples of gel and film had no difference in closure after treatment (0.26%), although after 24 h of treatment, both formulations in their lowest concentration presented closing values of 37.36% and 43.57% respectively, reaching 70.4% and 79.67% after 48 h and a complete scratch closure in 72 h. These results pointed out their potential use for wound healing properties, even more, if considering the phytochemical composition of the pinhão seed coat. For the values of molecules towards docking, the compounds with the highest negative interaction score values were quercetin and catechin, so it was used for molecular dynamics with the protein which presented a slightly moderate interaction with protein synthesis of Schwann cells, presenting compactness of the compound after 14 ns with an RMSD of 6 Å. The hydrogel formed in this work (PVA/Pinhão) can be applied in the most diverse areas such as wound treatment, production of controlled drug release systems, and scaffolds for tissue engineering, having enormous potential in the biomedical area.

## Figures and Tables

**Table 1:** Pinhão seed coat component in mg/kg and effective doses in cell lines, reference of values obtained from a previous work [30].

	Pinhão Seed Coat Component (mg/kg)	Effective Doses/Concentrations
Threo+erythro-guaiacylglycerol	54.64	IC_50_ (30.2 + 1.1 and 57.3 + 1.1μg mL^−1^) [48] 0.91 mM—agonistic rated (%) of 4.28, 0.64 and 6.04 [49]
Catechin	254.51	IC_50_ (0.43 μg mL^−1^) [50] IC_50_ (200; 400 and 600 μM) [51] IC_50_ (13.52 and 214.6 μg mL^−1^) [52]
Quercetin	70.24	24 h IC_50_ (113.65 μg mL^−1^) or 48 h (IC50 of IC_50_ (48.61 μg ml^−1^) [53] IC_50_ (1.5 μM) [54]
9-(Z)-Hexadecenoic acid	16	IC_50_ (3.125 to 100 µM) [55] IC_50_ (291~228 μM) [56]
9,12-(Z,Z)-Octadecadienoic acid	69.66	
9-(Z)-Octadecenoic acid	189.09	
24-Methyl-cholest-5-en-3β-ol (campesterol)	68.78	IC_50_ > 200 μM [57] 125 µM [58]
Beta-Sitosterol	449.72	16 μM [59] 90 μM [60] IC_50_ 24.7 μM [61]

**Table 2:** Molecular docking for all investigated compounds has two forms of interaction with the protein, two binding sites.

Compound	Affinity (kcal/mol)	Estimated Ki	Ki Units	Ligand Efficiency
Catechin	−7.6	2.69	uM	−0.36
Quercetin	−7.6	2.69	uM	−0.36
Guyacyl	−5.8	56.05	uM	−0.39
Oleic acid	−5.3	0.13	mM	−0.27
Campesterol	−6.8	10.37	uM	−0.23
Beta-sistosterol	−6.7	12.27	uM	−0.22
Quercetin	−7.6	2.69	uM	−0.36

## Data Availability

Not applicable.

## Supplementary Materials

The following supporting information can be downloaded at: https://www.mdpi.com/article/10.3390/polym14142776/s1, Supplementary Material S1: Discussion of the values found within the literature and the compound effect, as well as the EC50.

## Appendix A

**Table A1:** Pinhão compounds that interacted with PACAP+PAC1 key residues according to docking simulations.

Compound	H-Bond Interactions
Quercetin	**ARG12, ASN300, SER302, ASP301**
Catechin	**SER11**
Oleic acid	**CYS296**, *ASP298*
Guaiacylglycerol	**ARG12, ASP8, ASN375, ASP301, SER302**
Campesterol	--
Beta-sistosterol	--

**Bold and underlined ** are compounds bounded with PACAP, while bounded with PAC1 are in bold and unfavourable negative-negative in *italic*.

**Table A2:** H-bond values for the interaction between quercetin (QUE) and the conjugated protein PACAP+PAC1.

*DONOUR*			
*donour*	*acceptor*	*occupancy*	*protein*
***ASN375***	QUE	2.91%	PAC1
***TRP306***	QUE	2.27%	PAC1
***GLU374***	QUE	0.32%	PAC1
***LYS310***	QUE	1.29%	PAC1
***THR94***	QUE	1.29%	PAC1
***LYS378***	QUE	0.97%	PAC1
***ACCEPTOR***			
***donour***	***acceptor***	***occupancy***	***protein***
***QUE***	ASP8	2.27%	PACAP
***QUE***	PRO373	8.41%	PAC1
***QUE***	SER372	0.65%	PAC1
***QUE***	PHE371	0.65%	PAC1
***QUE***	ASN375	0.97%	PAC1
***QUE***	THR94	0.32%	PAC1
***QUE***	SER9	4.53%	PACAP
***QUE***	GLU93	6.47%	PAC1
***QUE***	LYS378	2.27%	PAC1
***QUE***	GLU385	0.97%	PAC1
