# Supplementary material for: Characterization of Gels and Films Produced from Pinhão Seed Coat Nanocellulose as a Potential Use for Wound Healing Dressings and Screening of Its Compounds towards Antitumour Effects
Source: Polymers (Basel). 2022 Jul 7;14(14):2776. doi: 10.3390/polym14142776 (PMC9315714; doi:10.3390/polym14142776)
Supplement: Supplementary file 1 [file polymers-14-02776-s001.zip › polymers-1798407-SI.pdf]

**Supplementary material S.1.**

Discussion of the values found within literature and the compound effect, as well as the EC50.

The difference in stereochemistry or substituent leads to guaiacylglycerol analogue compounds to exhibit different cytotoxic levels against cancer cell lines. For the threo+erythro-guaiacylglycerol, Lee et al. (2007) found the value of IC50(30.2 + 1.1 μg/mL) for the HCT-116 cancer cell lines and IC50(57.3 + 1.1μg/mL) for the cell lines HepG2 [1]. Wang et al. (2015) tested concentration of 0.9 mM for three different cells lines (MT1, 5-HT1A and 5-HT2C and found the agonistic rated (%) of 4.28, 0.64 and 6.04 respectively [2].

Quercetin and catechin are flavonoids that has been shown to have antioxidant, anti-inflammatory, anti-allergic, anti-viral and anti-cancer activities. Liu et all (2017) found that quercetin inhibited cell proliferation after cell treatment for 24 h (IC50 of 113.65μg/ml) or 48h (IC50 of 48.61μg/ml) [3]. Murakami et al., (2008) found the value of IC50 for quercetin 1.5 μM, [4]. Lin et al. (2021) compared catechin nanoemulsions and extracts to inhibiting DU-145 cell growth, results shows that nanoemulsions were more effective than extracts with the IC50 being 13.52 and 214.6 μg/mL, respectively, after 48 h incubation [5]. Cadoná et al. 2016 investigated the in vitro effects of catechin in cell proliferation on colorectal cancer (CRC) line HT-29 cells (IC50 0.43 μg/mL) [6]. Sun et al. 2020 found the inhibitory rate of catechin on the proliferation of A549 cells reached 19.76% at a concentration of 600 μmol·L −1 with 24 h incubation [7].

The main oleic acids found in the Pinhão seed coat are 9-(Z)-Hexadecenoic acid, 9,12-(Z,Z)-Octadecadienoic acid and 9-(Z)-Octadecenoic acid. Al-Mutairi et al. (2021) tested antineoplastic activity of gemcitabine (GM) and oxaliplatin (OXA) co-loaded into oleic acid-based solid lipid nanoparticle (OA-SLN) 3.125 to 100 μM. The oleic acid added significantly increased GM and OXA inhibition on the proliferation of A549 cells in all concentrations [8]. Jiang et al. (2017) founded that oleic acid effectively inhibited TSCC cell proliferation in a dose- and time-dependent manner. The IC50 values of oleic acid were 291~228 μM and 159~78 μM for CAL27 and UM1 cells, respectively [9].

Phytosterols (campesterol and beta-sitosterol) are naturally occurring compounds in plants, structurally similar to cholesterol and are natural anticancer agent. Yuan et al. (2020) assessed the inhibition of cholesterol absorption in Caco-2 cells by different phytosterols. The IC50 value of campesterol, β-sitosterol, stigmasterol, and stellasterol were > 200μM under the conditions tested [10]

. Bae et al. (2021) showed that campesterol activates cell death signals and cell death in human ovarian cancer cells. In the ES2 cells, the percentage of cells in the late apoptosis phase was increased to 2.6%,

5.4%, and 13.7% upon treatment with 25, 62.5, and 125 μM of campesterol, respectively, compared with that in the vehicle-treated control [11].

Awad et al. (2000) demonstrated that 16 μM Beta-Sitosterol as compared to 16 μM campesterol or 16 μM cholesterol inhibited MDA-MB-231 breast cancer cell growth by 66% after 3 days of treatment and 80% after 5 days of treatment. Another work in the literature demonstrated that 16 μM Beta-Sitosterol inhibited LNCaP tumour cell growth by 18-28% as compared to that of cholesterol after 5 days of treatment [12,13]. Vundru *et al.* 2013 found that the highest concentration (90 μM) of β-sitosterol for the A549 cells had a significant decrease in total cell number by 8.2% to 13.3% in 48 h and 3.4% to 13.7% in 72 h. For the MDA-MB-231 cells, 31% to 63% in 48 h and 40% to 50% in 72 h in cell growth inhibition. The increase in cell death was about 2% to 15% in 48 h and 1% to 4% in 72 h of ST treatments as compared to their respective controls [14]. Rajavel *et al.* 2018 showed that beta- sitosterol inhibits the growth of A549 cells without harming normal cells. In this work, strong growth inhibition was found after 72h time point with the IC50 value of 24.7μM [15].

**References**


1. Lee, D.-Y.; Song, M.-C.; Yoo, K.-H.; Bang, M.-H.; Chung, I.-S.; Kim, S.-H.; Kim, D.-K.; Kwon, B.-M.; Jeong, T.-S.; Park, M.-H.; et al. Lignans from the fruits of Cornus kousa Burg. and their cytotoxic effects on human cancer cell lines. *Arch. Pharm. Res.* **2007**, *30*, 402–407, doi:10.1007/BF02980211.

2. Wang, H.; Geng, C.-A.; Xu, H.-B.; Huang, X.-Y.; Ma, Y.-B.; Yang, C.-Y.; Zhang, X.-M.; Chen, J.-J. Lignans from the Fruits of Melia toosendan and Their Agonistic Activities on Melatonin Receptor MT1. *Planta Med.* **2015**, *81*, 847–854, doi:10.1055/s-0035-1546127.

3. Liu, Y.; Tang, Z.-G.; Lin, Y.; Qu, X.-G.; Lv, W.; Wang, G.-B.; Li, C.-L. Effects of quercetin on proliferation and migration of human glioblastoma U251 cells. *Biomed. Pharmacother.* **2017**, *92*, 33–38, doi:10.1016/j.biopha.2017.05.044.

4. Murakami, A.; Ashida, H.; Terao, J. Multitargeted cancer prevention by quercetin. *Cancer Lett.* **2008**, *269*, 315–325, doi:10.1016/j.canlet.2008.03.046.

5. Lin, Y.-H.; Wang, C.-C.; Lin, Y.-H.; Chen, B.-H. Preparation of Catechin Nanoemulsion from Oolong Tea Leaf Waste and Its Inhibition of Prostate Cancer Cells DU-145 and Tumors in Mice. *Molecules* **2021**, *26*, 3260, doi:10.3390/molecules26113260.

6. Cadoná, F.C.; Machado, A.K.; Azzolin, V.F.; Barbisan, F.; Dornelles, E.B.; Glanzner, W.; Gonçalves, P.B.D.; Assmann, C.E.; Ribeiro, E.E.; Cruz, I.B.M. da Guaraná a Caffeine-Rich Food

Increases Oxaliplatin Sensitivity of Colorectal HT-29 Cells by Apoptosis Pathway Modulation. *Anticancer. Agents Med. Chem.* **2016**, *16*, 1055–1065, doi:10.2174/1871520616666151217121138.

7. Sun, H.; Yin, M.; Hao, D.; Shen, Y. Anti-Cancer Activity of Catechin against A549 Lung Carcinoma Cells by Induction of Cyclin Kinase Inhibitor p21 and Suppression of Cyclin E1 and P–AKT. *Appl. Sci.* **2020**, *10*, doi:10.3390/app10062065.

8. Al-Mutairi, Ashwaq A., Mayson H. Alkhatib, H.M.G. Antitumor Activities of Co-loading Gemcitabine and Oxaliplatin into Oleic Acid-Based Solid Lipid Nanoparticle against Non-Small Cell Lung Cancer Cells. *Biointerface Res. Appl. Chem.* **2021**, *12*, 49–60, doi:10.33263/BRIAC121.049060.

9. Jiang, L.; Wang, W.; He, Q.; Wu, Y.; Lu, Z.; Sun, J.; Liu, Z.; Shao, Y.; Wang, A. Oleic acid induces apoptosis and autophagy in the treatment of Tongue Squamous cell carcinomas. *Sci. Rep.* **2017**, *7*, 11277, doi:10.1038/s41598-017-11842-5.

10. Yuan, L.; Zhang, F.; Jia, S.; Xie, J.; Shen, M. Differences between phytosterols with different structures in regulating cholesterol synthesis, transport and metabolism in Caco-2 cells. *J. Funct. Foods* **2020**, *65*, 103715, doi:10.1016/j.jff.2019.103715.

11. Bae, H.; Park, S.; Yang, C.; Song, G.; Lim, W. Disruption of Endoplasmic Reticulum and ROS Production in Human Ovarian Cancer by Campesterol. *Antioxidants* **2021**, *10*, 379, doi:10.3390/antiox10030379.

12. von Holtz, R.L.; Fink, C.S.; Awad, A.B. β-sitosterol activates the sphingomyelin cycle and induces apoptosis in LNCaP human prostate cancer cells. *Nutr. Cancer* **1998**, *32*, 8–12, doi:10.1080/01635589809514709.

13. Awad, A.B.; Gan, Y.; Fink, C.S. Effect of Β-Sitosterol, a Plant Sterol, on Growth, Protein Phosphatase 2A, and Phospholipase D in LNCaP Cells. *Nutr. Cancer* **2000**, *36*, 74–78, doi:10.1207/S15327914NC3601_11.

14. Vundru, S.S.; Kale, R.K.; Singh, R.P. β-sitosterol induces G1 arrest and causes depolarization of mitochondrial membrane potential in breast carcinoma MDA-MB-231 cells. *BMC Complement. Altern. Med.* **2013**, *13*, 280, doi:10.1186/1472-6882-13-280.

15. Rajavel, T.; Packiyaraj, P.; Suryanarayanan, V.; Singh, S.K.; Ruckmani, K.; Pandima Devi, K. β-Sitosterol targets Trx/Trx1 reductase to induce apoptosis in A549 cells via ROS mediated mitochondrial dysregulation and p53 activation. *Sci. Rep.* **2018**, *8*, 2071, doi:10.1038/s41598-018-20311-6.
